# Investigating the effects of global gene knockout of MrgF on motor performance and pain sensitivity in mice

**DOI:** 10.1186/s41065-025-00377-9

**Published:** 2025-03-03

**Authors:** Xuejiao Chen, Yan Chen, Runzhe Shu, Shunyuan Lu, Ming-Min Gu, Chunling Shen, Zhugang Wang, Xiaofang Cui

**Affiliations:** 1 Institute of Neuroscience and Brain science, Xiangyang Central Hospital, Affiliated Hospital of Hubei University of Arts and Science, Xiangyang, Hubei 441021 China; 2 State Key Laboratory of Medical Genomics, Research Center for Experimental Medicine, Rui-Jin Hospital, Shanghai Jiao Tong University School of Medicine, Shanghai, 200025 China; 3 Key Laboratory of Systems Biomedicine, Shanghai Center for Systems Biomedicine, Shanghai Jiao Tong University, Shanghai, 200240 China

**Keywords:** *MrgF*, Knockout mice, Cerebellum, DRG, Pain

## Abstract

Mas-related G protein-coupled receptors (Mrgs) are a subset of GPCRs linked to pain modulation. *MrgF* was identified as an orphan Mrg whose function and ligand remain unclear. In this study, in addition to its expression in the dorsal root ganglia (DRG), the primary afferent center that transmits pain, we identified dense expression of *MrgF*, particularly concentrated in the Purkinje cell layer of the mouse cerebellum. Given the the important role of Purkinje neurons in both motor modulation and non-motor modulation, including pain processing, we established a *MrgF* knockout mouse (*MrgF*^*−/−*^) model and performed a battery of behavioral tests to explore motor performance and assess pain-associated responses. *MrgF*^*−/−*^ mice exhibited no disturbances in coordination and motor balance during the rotarod, pole, balance beam, and treadmill tests, and normal cerebellar histology was retained. In hot plate assays, *MrgF*^*−/−*^ mice displayed reduced pain-related behavioral responses to thermal stimuli, although no significance differences were found in tail flick assays between *MrgF*^*−/−*^ and wild-type (wt) mice. Moreover, in formalin tests, *MrgF*^*−/−*^ mice also showed decreased chemical-induced nociception. This was accompanied by a downregulation in the expression levels of genes associated with nociceptive modulation, such as *c-fos*, *Runx1*, *Nav1.7*, *Nav1.8*, and *Nav1.9*, within the DRG of *MrgF*^*−/−*^ mice. Taken together, our findings suggest that MrgF may play a significant role in modulating pain sensitivity, thereby advancing the understanding of the functional characteristics of the Mrgs family.

**Supplementary Information:**

The online version contains supplementary material available at 10.1186/s41065-025-00377-9.

## Introduction

GPCRs typically consist of 300 to 1,000 amino acids and exhibit a distinctive seven α-helical transmembrane domain within their structures, predominantly located in mammalian cell membranes [1–3]. Members of the GPCR superfamily are distinguished by their interaction with a variety of endogenous ligands (e.g., hormones, neurotransmitters, growth factors) or sensory stimuli (e.g., light, taste, vision, pain), which activate coupled G-proteins to initiate a series of downstream signaling pathways, resulting in specific cellular responses [4, 5].

The Mas-related G protein-coupled receptors (Mrg receptors), members of the GPCR family, were initially identified as being specifically expressed in nociceptive sensory neurons, suggesting a significant role in pain modulation [6–8]. Beyond their expression in peripheral sensory system cells, Mrg receptors have also been detected in various other cell types and organs, including mast cells [9], enteric neurons [10], and multiple brain regions [11]. Based on sequence homology, the Mrg receptors are further divided into several subfamilies. Among them, the *MrgA*, *MrgB*, and *MrgC* subfamilies comprise up to fifty distinct members, which are exclusively expressed in rodents. Additionally, there are six kinds of single-copy gene families, including *MrgD*, *MrgE*, *MrgF* (*RTA*), *MrgG*, *MrgH*, and *Mas1*, displaying high homology between rodents and humans [12–16]. These six MRG members, predominantly orphan receptors, include MrgD, which is the most well-known and extensively characterized member. MrgD has been linked to the natural MasR ligand, angiotensin 1–7 (Ang 1–7) [16, 17]. However, no endogenous or exogenous ligands have been identified for MrgE and MrgF, unlike for other members of this receptor family, presenting a significant challenge in elucidating the physiological functions of these orphan receptors. A study using *MrgE* knockout mice suggests a potential role of the MrgE receptor in pain sensitivity [18]. In contrast, there has been limited research conducted on *MrgF*.

**Fig. 1:**
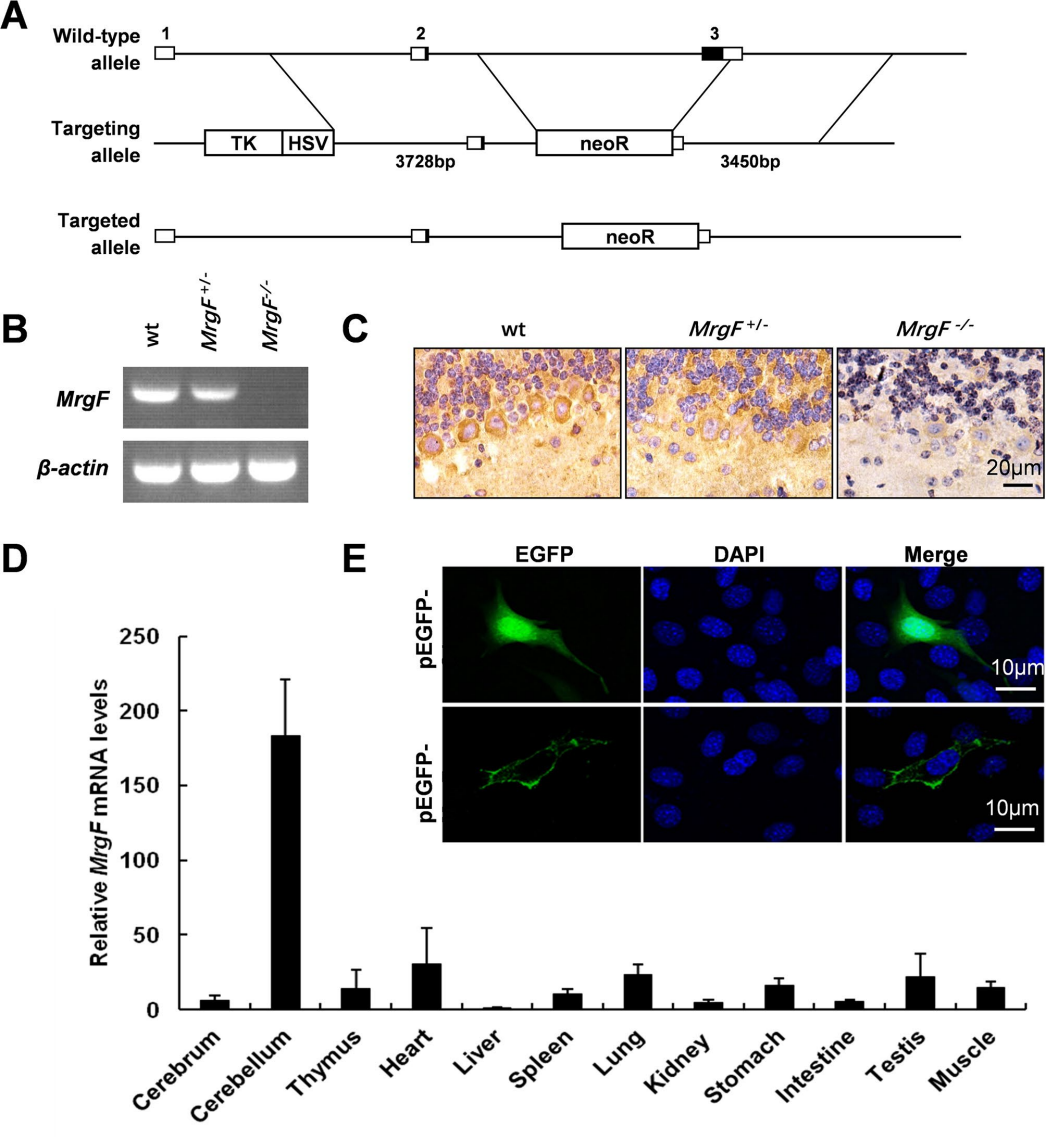
*MrgF* knock out mouse model generation and authentication. (**A**) *MrgF* gene knock-out strategy. The first row shows the wild-type *MrgF* gene structure, with squares 1, 2, 3 indicating the three exons, respectively. The start and stop codons are located in the second and third exons. The second row depicts the targeting vector structure, where the PGK-Neo cassette replases the third exon region of *MrgF* gene. (**B**) MrgF gene Expression in cerebellar tissue was quantified in three mouse genotypes using semi-quantitative RT-PCR. mRNA levels were normalized to β-actin, which served as an internal control. (**C**) MrgF expression in the cerebellum of mice from three genotypes was detected by immunohistochemistry (400×magnification). Positive signals appear brown (DAB) (*n* = 3). Scale bar = 20 μm. (**D**) The mRNA expression levels of *MrgF* expression was assessed by RT-PCR in various tissues, including the brain, cerebellum, thymus, heart, liver, spleen, lung, kidney, stomach, small intestine, testis and skeletal muscle. (**E**) Mouse MrgF cDNA was inserted into the pEGFP-C1 vector to creat the pEGFP-MrgF fusion plasmid, which was transfected into NIH3T3 cells alongside the empty vector. While the empty vector showed uniform expression, the fusion plasmid targeted the cell membranes. Confocal microscopy (400x magnification) revealed green fluorescence from EGFP and blue fluorescence from DAPI. Scale bar = 10 μm

**Fig. 2:**
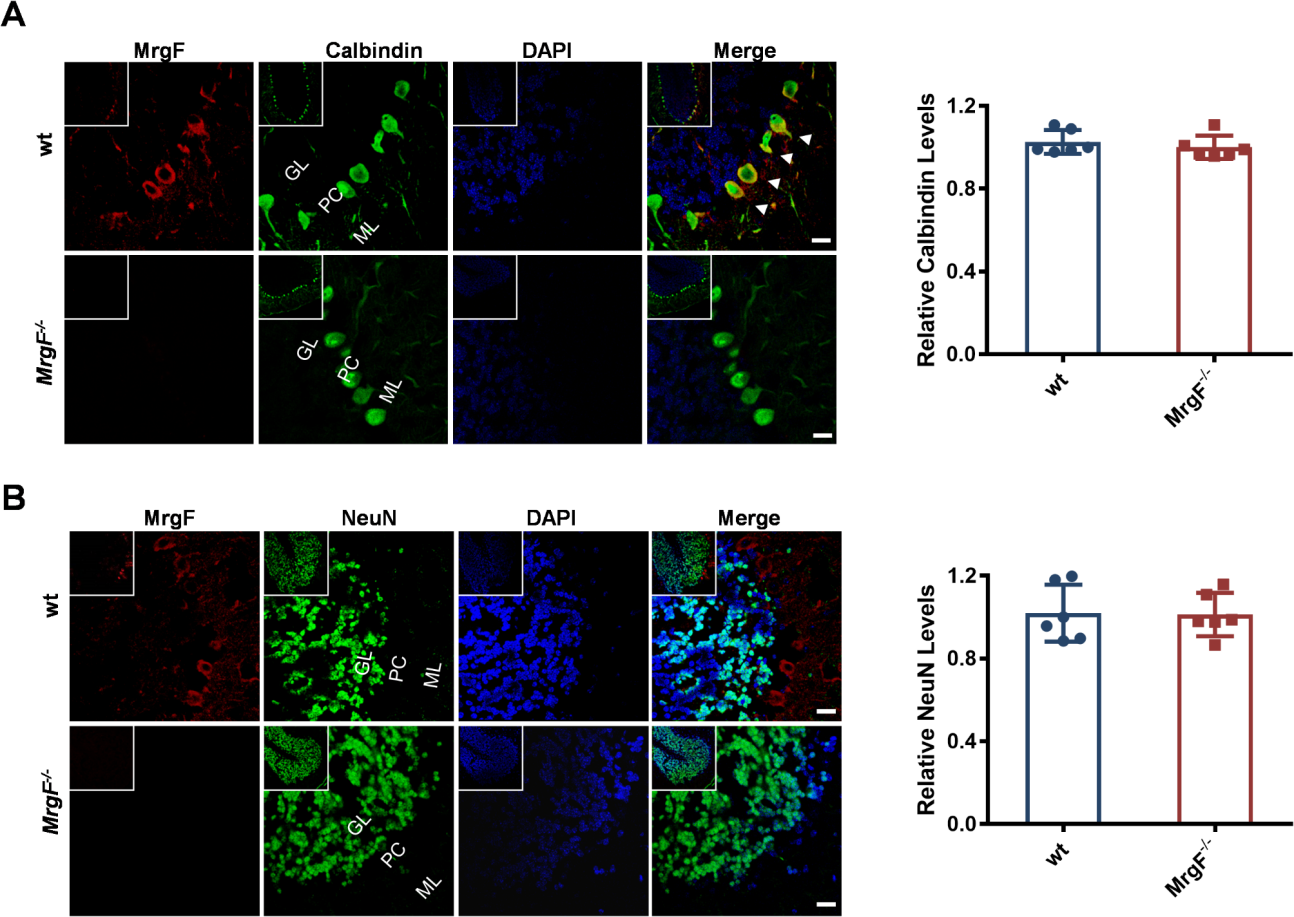
The distribution of MrgF in the cerebellum. (**A**) Distribution of MrgF in Purkinje cells of wt and *MrgF*^*−/−*^ mice. Confocal laser scanning microscopy was used to analyz the distribution of MrgF in Purkinje cells of wt and *MrgF*^*−/−*^ mice. The images show MrgF (red), Calbindin (green), and DAPI (blue). (**B**) Distribution of MrgF in Granular cells of wt and *MrgF*^*−/−*^ mice cerebellum. The distribution of MrgF in the granule cells of the cerebellum from wt and *MrgF*^*−/−*^ mice was examined using confocal laser scanning microscopy. The images show MrgF (red), NeuN (green), and DAPI (blue). GL, granule cell layer; ML, molecular layer; PL, Purkinje cell layer. Scale bar = 20 μm. Results are shown as mean ± s.d. for each panel

**Fig. 3:**
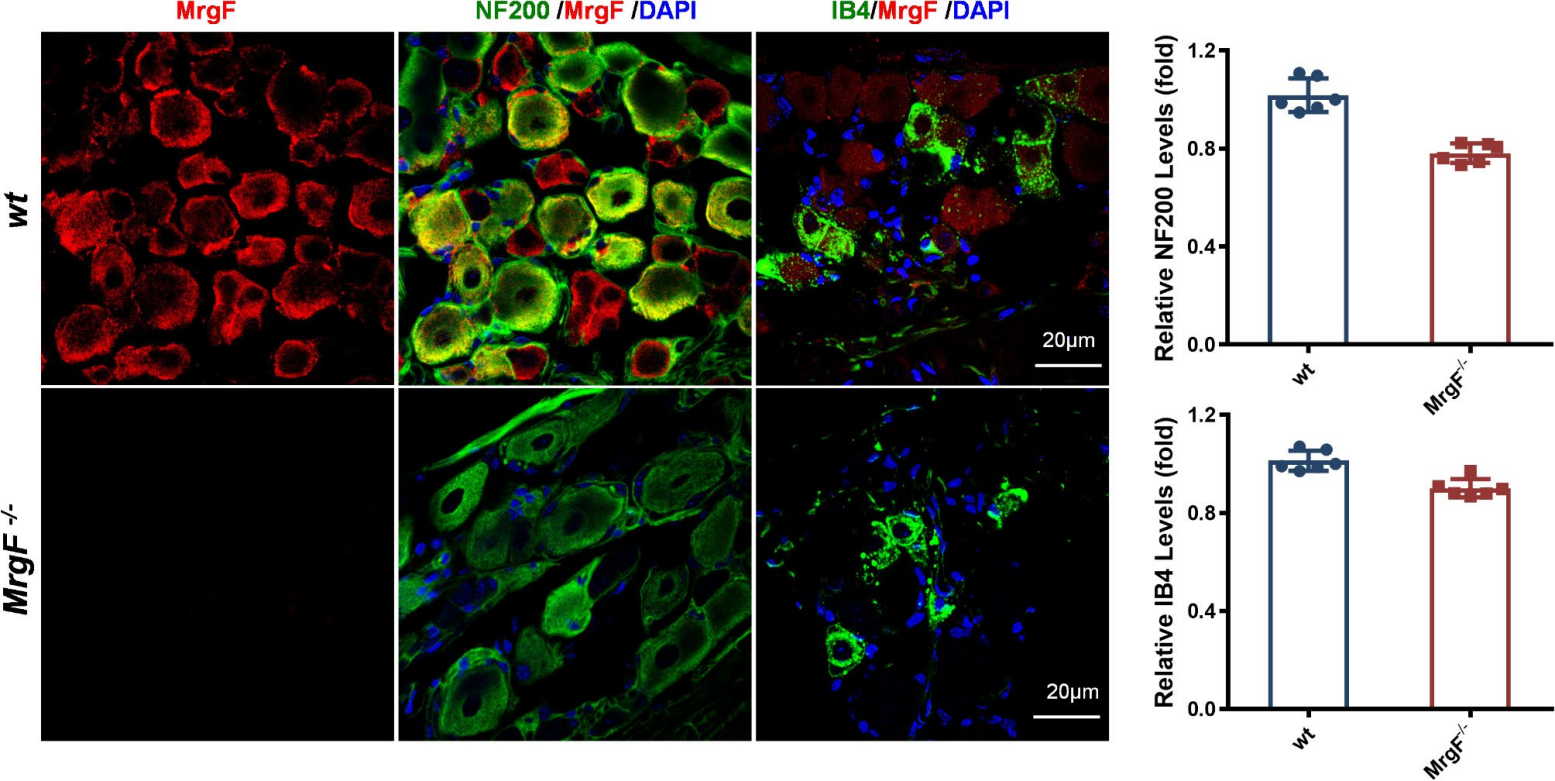
MrgF is extensively distributed in DRG. Immunofluorescence confocal imaging, red indicates MrgF; green indicates NF200, IB4; blue indicates DAPI; Original magnification, × 400. Scale bar = 20 μm. Results are shown as mean ± s.d. for each panel

It has been previously noted that MrgF exhibits significantly higher levels of abundance in the rat cerebellum compared to other family members. However, until recently, the cellular specificity of MrgF expression and its role in the cerebellum remained unexplored. In this study, considering that the specific expression of a gene in a particular organ is often interpreted as indicating that the gene has a specific function, we developed an *MrgF* gene knockout (*MrgF*^*−/−*^) mouse model to address the limitations posed by the absence of specific ligands (Fig. 1). Our findings demonstrated the localization of the MrgF receptor on Purkinje cells within the cerebellum, which are implicated in both motor and non-motor functions, including pain processing. Successful knockout of the *MrgF* gene has been verified in the cerebellum and DRG cells of *MrgF*^*−/−*^ mice (Purkinje cells, Fig. 2; DRG, Fig. 3). The mass of the cerebellum and cerebrum normalized to body weight showed no significant difference between *MrgF*^*−/−*^ mice and their wt littermates, nor did the blood cytology and metabolism of mice (Supplementary Fig. S1; Tables S2 & S3).

The effects of MrgF deletion on the mouse model were evaluated through a series of behavioral tests assessing motor performance, as well as thermal- and formalin -induced pain. Our findings show that the absence of MrgF does not impact locomotor activity or motor coordination. Further investigation suggests that a deficiency in MrgF may have a slight impact on anxiety levels and could potentially decrease nociception induced by thermal and formalin stimuli in mice. Furthermore, the study examined the effects of deleting MrgF on the expression of pain-related genes in sensory neurons, including other Mrg receptors. Given the limited understanding of the biological function of MrgF, these findings regarding its role in both the central and peripheral nervous systems provide initial evidence for future research aimed at elucidating its mechanisms in pain modulation.

## Materials and methods

### Ethics statement

Animal experiments were conducted in accordance with the Society’s Policies on the Use of Animals and Humans in Neuroscience Research. The research protocol received approval from the Institutional Animal Care and Use Committee of the Shanghai Research Center for Model Organisms.

### Animals

A targeting vector was engineered by substituting a 3487 bp fragment of the mouse MrgF genomic region, encompassing exon 3, with a 1904 bp phosphoglycerate kinase-neomycin resistance cassette (PGK–Neo cassette) to facilitate positive selection. Additionally, an external herpes simplex virus-1–thymidine kinase cassette (HSV-TK cassette) was incorporated for negative selection. The mutant mice were maintained on a mixed 129 Sv/C57BL/6 genetic background under specific pathogen-free (SPF) conditions. They were housed at a constant room temperature of 22–24 °C, with a 12-hour light/dark cycle (lights on at 6 AM and off at 6 PM), and had ad libitum access to a standard chow diet and water.

### qRT- PCR

Both reverse transcription PCR and quantitative real-time PCR (qRT-PCR) were employed to evaluate the relative expression levels of MrgF in mouse tissues. The quantification of Mrg family members, as well as genes implicated in sensory neuron development and function, was conducted in cerebellum and DRG tissues from adult wt and *MrgF*^*−/−*^ mice using qRT-PCR analysis. In three independent experiments, all samples were analyzed and normalized to β-actin expression. Detailed information regarding all targeted genes is provided in Supplementary Table S1.

### Isolation of the dorsal root ganglia

DRG were isolated from young adult mice aged 2 to 3 months. Both wt and *MrgF*^*−/−*^ mice used in these experiments were littermates. Following anesthesia with 1% (v/v) sodium pentobarbital in saline, the spinal column was removed. An incision was made to access the lumbar, cervical, and thoracic DRGs, which were trimmed in cold and sterilized phosphate-buffered saline (PBS). The ganglia were transferred to a conical tube, washed with sterilized PBS, and stored at -80 °C for use within two weeks. For cases requiring frozen section analysis, intracardiac perfusion of the mice was conducted, followed by fixation in 4% paraformaldehyde (PFA). The L4-L5 dorsal root ganglion was then harvested for frozen sectioning at a thickness of 8 μm.

### Histological analysis

According to our previous reports [19, 20], immunohistochemistry, histology, and immunofluorescence were performed. The Cerebellum and DRG were dehydrated and subsequently embedded in paraffin. Paraffin blocks were sectioned at a thickness of 10 μm. The following antibodies and their respective dilutions were utilized: rabbit anti-MrgF (1:200; Abcam), mouse anti-Calbindin (1:3000; Sigma), mouse anti-NeuN (1:200; Millipore), and mouse anti-NF200 (1:100; Millipore). IB4 binding was detected using Griffonia simplicifolia isolectin GS-IB4-Alexa 488 (1:200; Molecular Probes). Negative controls were prepared by omitting the primary antibodies. The specificity of the secondary antisera was verified by performing interference controls in which the primary antisera were omitted. The fluorescence intensity is calculated using Image J software.

### Transmission electronic microscopy

Transmission electron microscopy (TEM) was performed at the Laboratory of Electron Microscopy at Shanghai Jiao Tong University School of Medicine following established protocols. Briefly, cerebellums from wt and *MrgF*^*−/−*^ mice were fixed in glutaraldehyde and osmium tetroxide, embedded in 10% (w/v) gelatin, dehydrated using sucrose, and subsequently frozen in liquid nitrogen. TEM images were obtained after preparing cryosections of 50 nm thickness using an ultramicrotome (Ultra-Cut UCT/Leica EMFCS), staining the samples with uranyl acetate and methylcellulose, and examining them under the microscope.

### Behavioral overview

All behavioral assays were performed by an experimenter blinded to the genotypes of the subjects. Male mice, aged 2–3 months and weighing between 20 and 30 g, were used in the study. Prior to the behavioral tests, all mice were acclimated to the testing environment for at least of 30 min. The mice were housed in groups of 4–5 per cage in a vivarium maintained under a 12-hour light/dark cycle. All behavioral tests were conducted in the afternoon and included the open field test (OFT), elevated plus maze (EPM), rotarod test, pole test, and traversing beam test, which were carried out according to previously established protocols [19, 20].

#### Treadmill test

Within the enclosure, a mouse treadmill with a headroom of 4.4 and 5 cm width was installed. The running surface length, measured from the top of each roller, was 25.4 cm, resulting in an approximate volume of 2 L. During the treadmill test, each mouse underwent a training regimen consisting of five minutes of running at a constant speed of 8 m per minute, conducted every two days for three sessions. Following this acclimation period, all mice were subjected to a protocol involving incremental running speeds, starting at 8 m per minute and increasing by 2 m per minute every 15 min, up to a maximum of 20 m per minute, on a 0° incline. The trial ended when the mouse exhibited signs of exhaustion. Latency time and running distance were recorded.

#### Hot plate test

The hot plate test was conducted at a temperature of 55 °C using the Ugo Basile Hot/Cold Plate 55,100 apparatus. The latency period for the hind paw licking response was recorded. To prevent tissue damage, a cut-off time of 30 s was set for the 55 °C condition. Animals that did not respond within the cut-off time were removed from the test and assigned a latency score equal to the cut-off time. Female mice used in this study were 2–3 months old and weighed between 20 and 30 g.

#### Tail flick test

In the tail flick test, a light beam was directed at the distal one-third of the mouse’s tail, starting a timer. The timer was stopped when the mouse flicked its tail, and the recorded latency was used as a measure of pain threshold (Ugo Basile Tail Flick 57360). Each subject underwent three trials, with 30-minute intervals between trials. The maximum allowable latency was set at 30 s to prevent tissue damage.

#### Formalin test

Each mouse was acclimated to a transparent plastic cage at least 30 min prior to the administration of formalin to ensure environmental adaptation. Subsequently, a 25 µL solution of 5% (v/v) formalin in saline was injected into the plantar surface of the right hind paw. Pain-related behavior was evaluated by recording the number of flinches (rapid paw shaking) of the injected paw. The total number of hind paw flinches was quantified during two distinct observation periods: 0 to 5 min and 15 to 30 min post-formalin injection.

### Statistical analysis

The statistical analysis involved presenting data as the mean ± standard deviation and utilizing ANOVA to analyze differences among group means. Unless otherwise specified, comparisons between two variables across groups were performed using a two-tailed Student’s t-test. A *p*-value of less than 0.05 was considered statistically significant.

## Results

### Establishment and identification of *MrgF*^*−/−*^ mice

The *MrgF* gene knockout mouse model was established through the targeted deletion of exon 3 (Fig. 1A). The absence of *MrgF* mRNA and protein expression in the cerebellum was verified using semi-quantitative reverse transcription polymerase chain reaction (RT-PCR) and immunohistochemical analysis (Fig. 1B and C). The tissue expression profile of *MrgF* was assessed, revealing mRNA expression levels in various tissues, including the cerebrum, cerebellum, thymus, heart, liver, spleen, lung, kidney, stomach, intestine, testis, and skeletal muscle, as determined by RT-PCR. MrgF exhibits high expression levels in the cerebellar tissue of mice (Fig. 1D). MrgF is characterized as a membrane surface expression protein (Fig. 1E).

The *MrgF*^*−/−*^ mice were found to be viable, normal in size, and fertile, with no gross developmental abnormalities observed (Supplementary Fig. S1; Tables S2 & S3).

### Distribution pattern of *MrgF* in the cerebellum of mice

To more precisely delineate the distribution patterns within cerebellar regions, immunofluorescence staining was conducted using Calbindin, a specific marker for Purkinje cells, and NeuN, a marker for granule neurons, on cerebellar sections from adult wt and *MrgF*^*−/−*^ mice. Our observations revealed that MrgF predominantly localized to the membranes of Calbindin-positive Purkinje cells (Fig. 2A), and was absent in the NeuN-positive granule cell layer of the cerebellum (Fig. 2B).

To investigate whether the morphology of cerebellum is affected due to the deficiency of *MrgF*, we conducted histological and transmission electron microscopy (TEM) analysis and observed no discernable morphological changes in the cerebellum of *MrgF*^*−/−*^ mice (Supplementary Fig. S2). These findings collectively suggest that the expression of MrgF is particularly concentrated in the Purkinje cell layer of the cerebellum within the central nervous system, warranting further investigation into the biological role of MrgF in cerebellar function.

### Effect of *MrgF* deficiency on motor activity and exploratory behaviors in mice

**Fig. 4:**
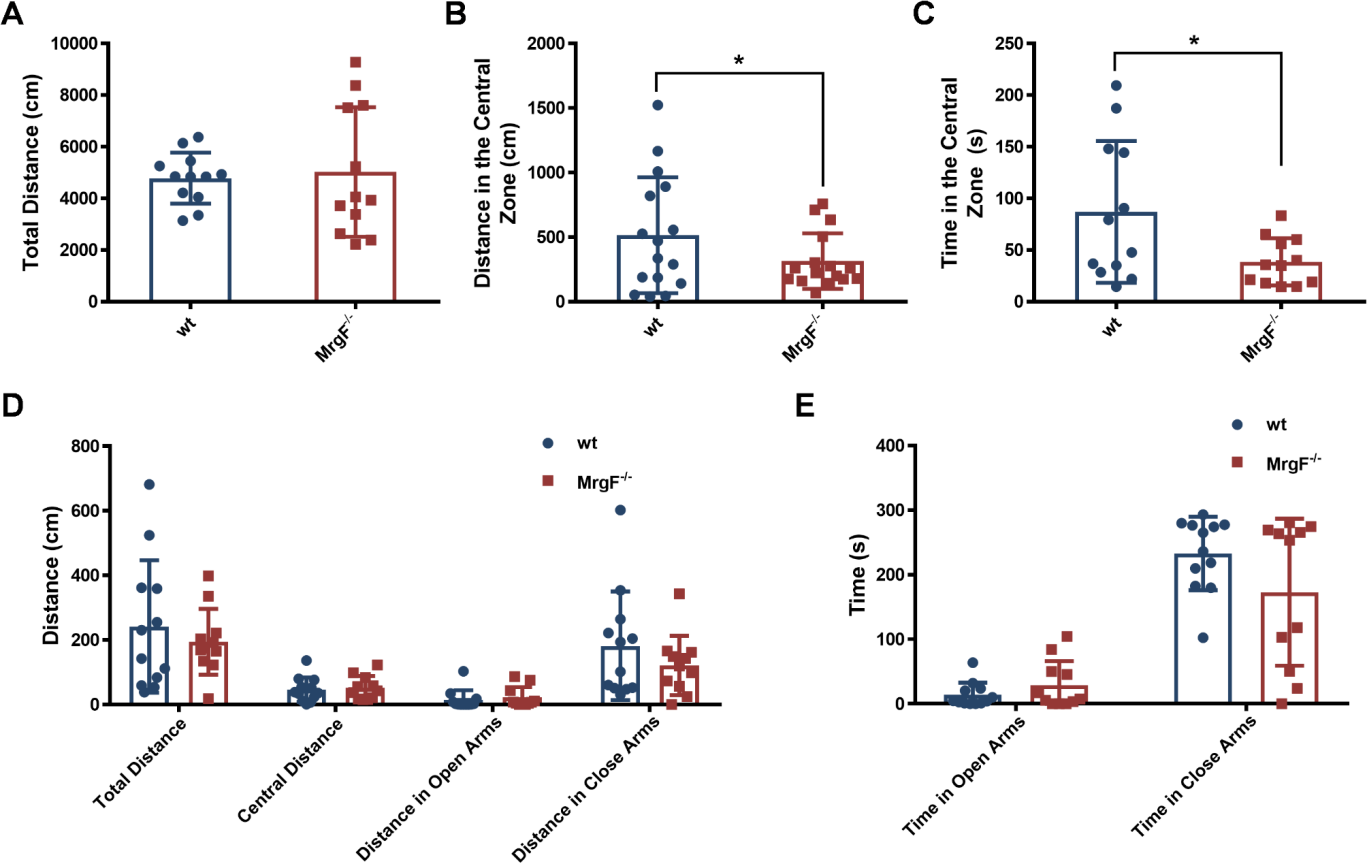
*MrgF* deficiency impacts the exploratory behaviors and does not affect locomotor behaviors in mice. (**A, B, C**) The total travel distance (**A**), distance in the central zone (**B**) and time spent in the central zone (**C**) were compared between wt and *MrgF*^*−/−*^ mice in the open field test. (**D, E**) The total travel distance (**D**) and the time (**E**) spent in the open and closed arms were compared between the two genotypes in the elevated plus maze (EPM) test. Results are shown as mean ± s.d. for each panel (Statistical comparrisions were made with the wt group, *p < 0.05)

To investigate potential changes in locomotor activity, exploratory behavior, and anxiety-related behavior in insecure area due to *MrgF* deficiency, we subjected *MrgF*^*−/−*^ and wt mice to the open-field test and elevated plus maze analysis. *MrgF*^*−/−*^ mice exhibited normal locomotion activity, as indicated by the total distance traveled compared to their wt littermates (Fig. 4A). However, the marginally reduced distances traveled and delayed entrance times into the center zone during the open-field test (Fig. 4B and C), suggest that *MrgF* deficiency may impede exploratory behavior in mice. Conversely, in the elevated plus maze test, *MrgF*^*−/−*^ mice did not exhibit a significant difference; they explored the open arms with similar frequency and spent comparable amounts of time in these areas as the wt group (Fig. 4D and E). These findings suggest that *MrgF* deficiency does not significantly affect locomotor activity in mice.

### Effect of *MrgF* deficiency on motor coordination and balance related behaviors in mice

**Fig. 5:**
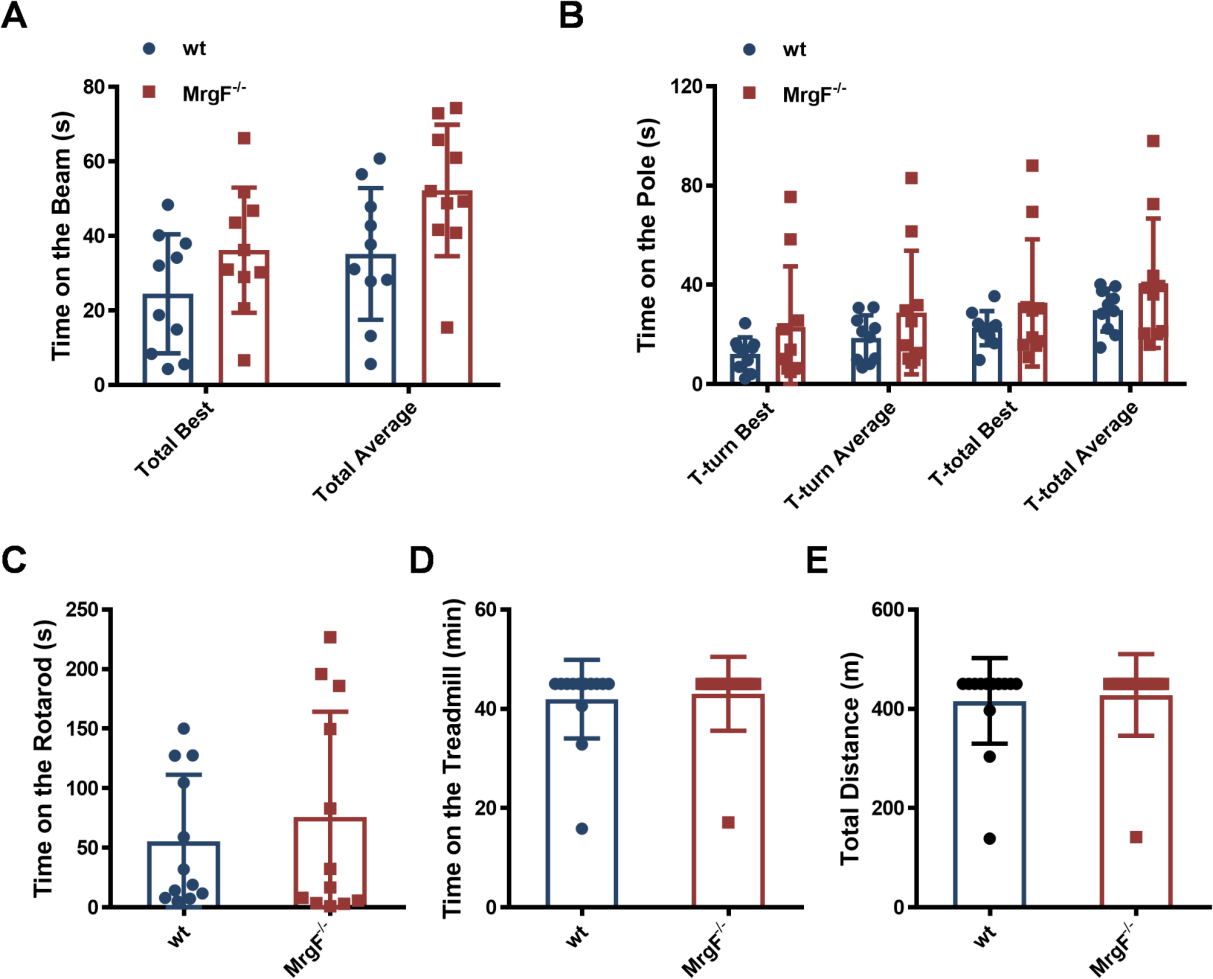
*MrgF* deficiency has no effect on motor coordination and balance related behaviors in mice. (**A**) Beam traversing test: Time spent crossing the balance beam. (**B**) Pole test: T-turn time and T-total time in the pole test. (**C**) Rotarod-test: Mean time on the Rotarod. (**D, E**) Treadmill test: Time spent on the treadmill and total distance covered before falling. Results are shown as mean ± s.d. for each panel

Considering that MrgF expression is primarily localized in the Purkinje cells of the cerebellum, which play a crucial role in coordination and motor function regulation, we undertook a comprehensive series of assessments to evaluate potential motor or functional deficits in *MrgF*^*−/−*^ mice. The assessments included the traversing beam test (Fig. 5A), pole test (Fig. 5B), rotarod test (Fig. 5C), and treadmill test (Fig. 5D and E). The findings consistently demonstrated no significant differences in motor coordination and balance between *MrgF*^*−/−*^ mice and their wt counterparts.

### Effect of *MrgF* deficiency on pain sensitivity in mice

**Fig. 6:**
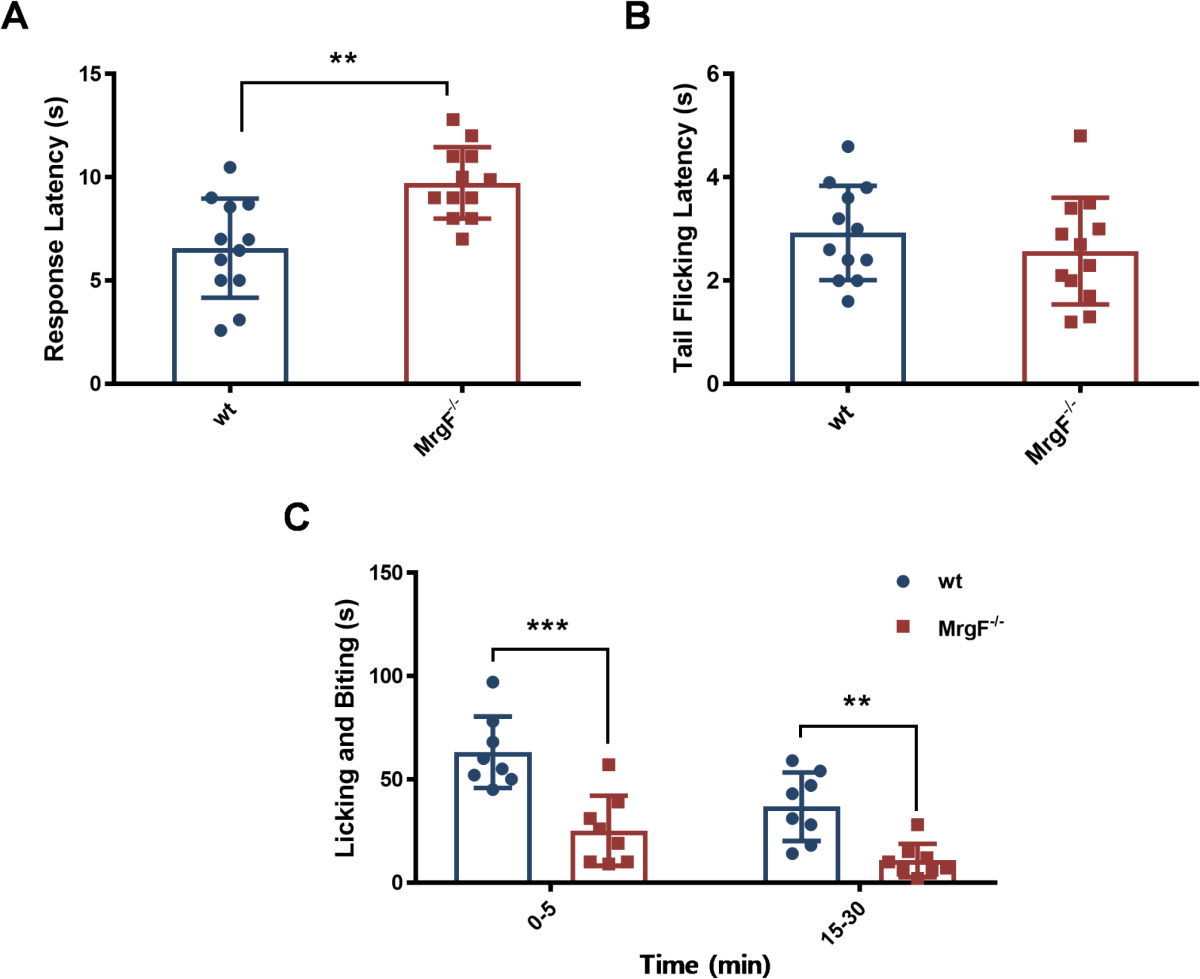
*MrgF* deficiency increases significant tolerance in pathological nociception. (**A**) Hot plate test: Latency to licking of the hind paw (seconds). (**B**) Tail flick test: Time (seconds) between the start of light beam stimulation and the flick response. (**C**) Formalin test: Total numbers of hind paw flinches observed during the first 0 to 5 minutesand the 15–30 min after formalin injection (Y-axis indicates flinch counts). Results are shown as mean ± s.d. for each panel (Statistical comparrisions were made with the wt group, **p < 0.01, ***p < 0.001)

Thermal and chemical stimuli are the two main neurobiological pathways inducing acute pain [21]. To investigate the potential role of *MrgF* in pain, we first performed a thermal stimuli-induced pain test to compare *MrgF*^*−/−*^ and wt mice. As shown in Fig. 6A, in the hot plate test (HPT), *MrgF*^*−/−*^ mice exhibited significantly lower pain sensitivity than wt mice (Fig. 6A). However, there was no significant difference in the latency time in the tail flick test (TFT) between *MrgF*^*−/−*^ and wt mice (Fig. 6B). These data suggest that *MrgF* deficiency might impact pain reflexes in response to thermal stimulus.

To further explore the role of *MrgF* in pain perception, we used formalin-induced pain model. Results are shown as a duration of the licking response to formalin stimulation in the rear paw of *MrgF*^*−/−*^ mice was significantly shorter than that observed in wt mice during the first (0 to 5 min) and the second (15 to 30 min) phases (Fig. 6C), indicating increased tolerance to formalin-induced pain. These finding demonstrate that *MrgF* deficiency alleviate formalin-evoked pain behavior.

### Distribution of *MrgF* in the DRG of mice

The composition of DRG neurons includes large and small neurons. Both large and small neurons are involved in the perception of pain and temperature within the DRG. We employed IB4 and NF200 antibodies for co-localization with the MrgF protein via immunofluorescence. MrgF was expressed not only in non-peptidergic IB4-positive small neurons but also in NF200-labeled large neurons (Fig. 3).

### Effect of *MrgF* deficiency on the expression of pain-related molecules in mice cerebellum and DRG

**Fig. 7:**
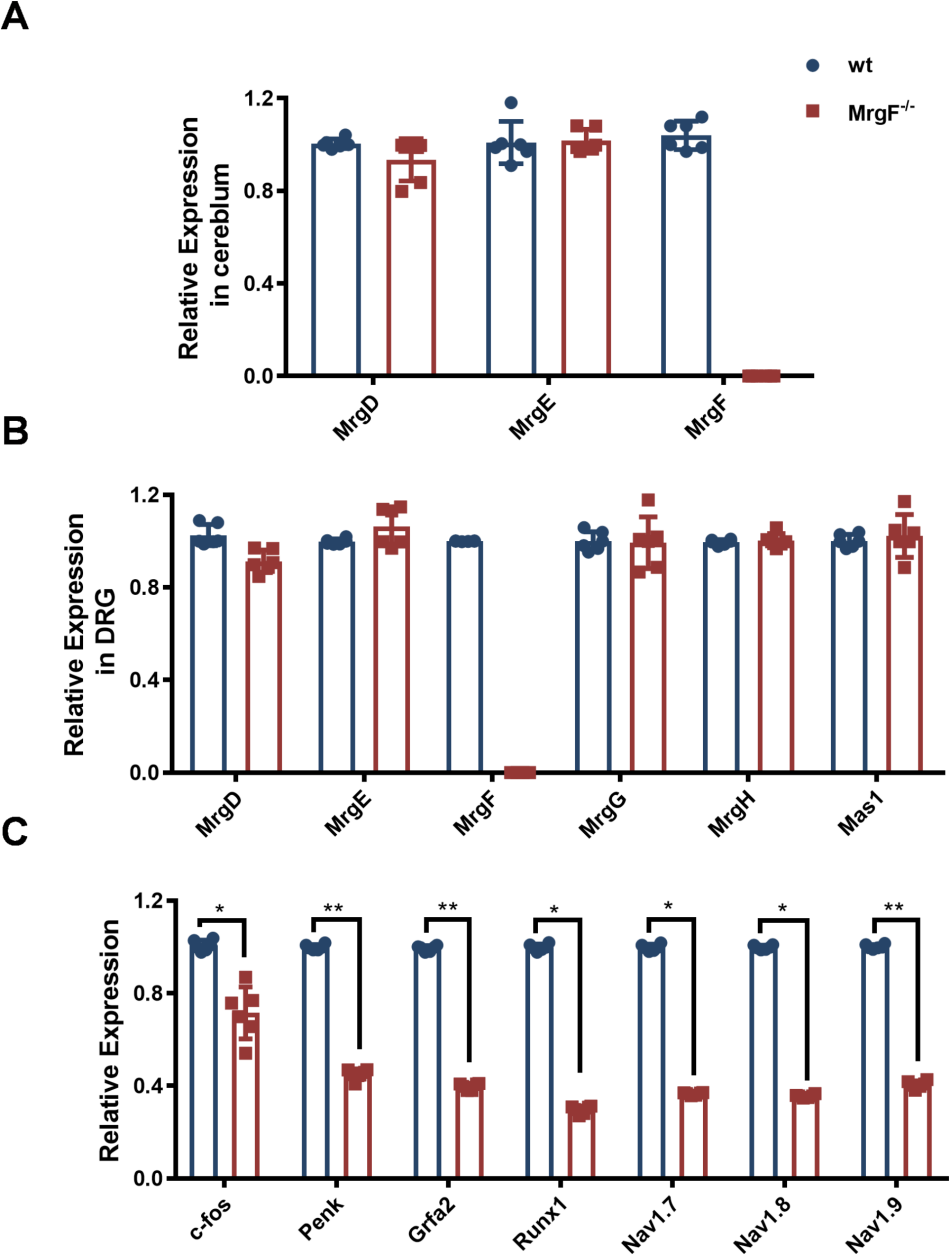
qRT-PCR analysis of *Mrgs* expression and genes associated with sensory neuron function. (**A**) qRT-PCR detection of *MrgD*, *MrgE*, and *MrgF* gene expression in the cerebellum of wt and *MrgF*^*−/−*^ mice. (**B**) qRT-PCR detection of *MrgD*, *MrgE*, and *MrgF* gene expression in the DRG of wt and *MrgF*^*−/−*^ mice. (**C**) qRT-PCR detection of *Gfrα2*, *Runx1*, *Nav1.7*, *Nav1.8*, and *Nav1.9* gene expression in the DRG of wt and *MrgF*^*−/−*^ mice. Results are shown as mean ± s.d. for each panel (Statistical comparrisions were made with the wt group, *p < 0.05, **p < 0.01)

*MrgD*, *MrgE* and *MrgF* are clustered on chromosomes 7 of the mouse genome. It has been reported that *MrgD* and *MrgE* form a heterodimer, influencing intracellular signal transmission [22]. Consequently, we initially assessed the mRNA expression levels of *MrgD*, *MrgE* and *MrgF* in the cerebellum, as well as the mRNA expression levels of *MrgD*, *MrgE*, *MrgF*, *MrgG*, *MrgH*, and *Mas1* in the DRG utilizing qRT-PCR, which indicated that there was no statistically significant difference in the expression levels of these family members between *MrgF*^*−/−*^ and wt mice (Fig. 7A & B). Furthermore, our findings reveal that *MrgF*^*−/−*^ mice exhibit a significant reduction in the expression of c-fos, Penk, *Gfrα2*, *Runx1*, *Nav1.7*, *Nav1.8*, and *Nav1.9* in the DRG (Fig. 7C). These results suggest that *MrgF* deficiency leads to reduced expression of genes critical for nociceptive sensory neurons, potentially explaining the sensitivity phenotype observed in *MrgF*^*−/−*^ mice.

## Discussion

MrgF, a less understood member of the Mrgs family, is mainly expressed in the cerebellum and DRG, with our study highlighting its dense presence in cerebellar Purkinje cells. Using a global deletion mouse model, we found that MrgF deficiency does not alter Purkinje cell number or morphology, nor does it impact motor balance or coordination. However, *MrgF*^*−/−*^ mice exhibited reduced pain responses to thermal stimuli and formalin test, indicating a role for MrgF in pain modulation. Additionally, MrgF deficiency was associated with decreased expression of genes vital for sensory neuron development and function in the DRG.

In our study, we found that *MrgF* expression was observed to be widespread in the DRG, with localization in both NF200-positive large-diameter neurons and IB4-positive small-diameter neurons (Fig. 3). Previous research has shown that sensory neuron-specific Mrgs are selectively expressed in the DRG and act as markers for non-peptidergic neurons [6, 15, 23]. Our findings reveal that, in contrast to other genes within the same family, MrgF exhibits a more extensive distribution. There was indeed a down-regulation trend of the NF200 and IB4 immunostaining. However, the relative expression of NF200 and IB4 did not vary significantly between the *MrgF*^*−/−*^ and wt mice group. Nonetheless, employing additional molecular markers to conduct more targeted assays would further elucidate the role of MrgF.

Cerebellar Purkinje cells are the most important information-processing elements in the cerebellum [24]. Although the cerebellum has traditionally been associated with motor information processing, other roles, including pain processing, have been proposed in recent years [25–28]. Using H&E staining and TEM analysis, no significant structural changes were observed in the cerebellum of *MrgF*^*−/−*^ mice at the histological level (Supplementary Fig. 2). Moreover, motor phenotypes revealed no differences in several behavioral tests between *MrgF*^*−/−*^ and wt mice. However, the specific role of Purkinje cells in cerebellar in pain processing remains elusive. The classification of sensory DRG neurons encompasses large myelinated Aβ fibers, thinly myelinated Aδ fibers, and small unmyelinated C fibers. At present, the involvement of cerebellar Purkinje cells in peripheral nociception remains inadequately understood. Nonetheless, it has been established that Aδ- and C-fiber signals are transmitted to Purkinje cells within the cerebellum [25, 29–31], and that nociceptive somatosensory and visceral signals can induce Purkinje cell firing [26, 32, 33]. We hypothesize that the *MrgF* gene may play a crucial role in the signaling processes of Aδ- and C-fibers, which are responsible for transmitting nociceptive signals from peripheral nociceptors to cerebellar Purkinje cells. This hypothesis necessitates further investigation to elucidate the specific challenges and underlying mechanisms involved. Additional studies are required to ascertain whether the dense localization of MrgF in Purkinje cells is linked to the cerebellum’s role in pain processing.

In this study, we employed animal models to investigate acute pain induced by thermal and formalin-evoked pain behavior. In hot plate assays, *MrgF*^*−/−*^ mice exhibited diminished pain-related behavioral responses to thermal stimuli; however, no significant differences were observed in tail flick assays between *MrgF*^*−/−*^ and wt mice. It is crucial to utilize female mice for the hot plate test due to the anatomical structure of male mice, wherein the proximity of the scrotal skin to the metal hot plate may yield confounding results, as the testicular region is more heat-sensitive than the paws. Consequently, we exclusively utilized female mice for the hot plate test to address the limitations associated with the model. In contrast, the tail flick test, which included male mice, demonstrated no significant difference in latency time between *MrgF*^*−/−*^ and wt mice. Given the specific sex requirements for the hot plate experiment, it remains unclear whether MrgF deficiency influences pain-related behavior in male or female mice. Further investigation is necessary to ascertain whether MrgF affects pain sensitivity differently based on sex. Additionally, the future implementation of the Hargreaves assay, as described by de Sousa Valente et al. [34], is warranted to clarify the precise role of MrgF in the thermal pain model. However, MrgF deficiency significantly attenuated formalin-evoked pain behavior in mice, which further suggest that MrgF may play a significant role in the transduction of pain signals in somatosensory DRG neurons. We propose that MrgF deficiency impairs the function of both small- and large-sized DRG neurons in conveying pain signals. The primary limitations of our study on the regulation of pain sensitivity by MrgF include the paucity of validation studies and data related to mechanical noxious stimulation and models involving more stimulated conditions. It is essential to evaluate the effects on MrgF expression to elucidate its role under various stimulated conditions, extending beyond the baseline constitutive knockout scenarios.

Among the members of the single-copy gene family Mrgs, *MrgD*, *MrgE*, and *MrgF* are expressed in the cerebellum. Our analysis revealed that the expression levels of *MrgD* and *MrgE* did not exhibit significant compensatory alterations in the cerebellum of *MrgF*^*−/−*^ mice. Although no statistically significant differences were detected among the *MrgD*, *MrgE*, *MrgG*, *MrgH*, *Mas1*, there was a slight trend towards increased expression of MrgE and decreased expression of MrgD in the DRG of *MrgF*^*−/−*^ mice. Investigating this receptor family under stimulated or challenged conditions is of significant interest, as further research and a detailed understanding are essential for the specific functions of Mrgs in pain perception.

In our study, we observed a down-regulation in the expression levels of genes associated with nociceptive modulation in the DRG of *MrgF*^*−/−*^ and wt mice. Notably, the expression of the *c-fos* gene, which is significantly associated with pain, was reduced. This reduction in *c-fos* transcription corresponded with a downstream decrease in the expression of the *Penk* gene. The transcription factor Runx1 is identified as a critical regulator of Mrg receptors’ expression and plays a significant role in neuronal specialization during the late development of DRG neurons [35]. Mice deficient in Runx1 demonstrate distinct impairments in thermal and neuropathic pain responses [36]. The voltage-gated sodium channel isoforms Nav1.7, Nav1.8, and Nav1.9 are primarily implicated in pain signaling [37], which can also be regulated by the Runx1. The observed down-regulation in mRNA levels of c-fos, Penk, Gfrα2, Runx1, Nav1.7, Nav1.8, and Nav1.9 may provide an additional explanation for the increased pain tolerance observed in mice. Future studies should conduct transcriptome-wide association analyses, incorporating both the cerebellum and DRG under more stimulated conditions, to elucidate the role of MrgF.

In summary, the present findings demonstrate distribution of the MrgF receptor in the Purkinje cell layer of the cerebellum and in DRG neurons, highlighting its potential role in pain perception. These results motivate further research into the function of the Mrgs family in pain modulation and may provide potential drug targets for pain therapy.

## Electronic supplementary material

Below is the link to the electronic supplementary material.

Supplementary Material 1

Supplementary Material 2

## Data Availability

No datasets were generated or analysed during the current study.
