# Supplementary figures and images for: Investigating the effects of global gene knockout of MrgF on motor performance and pain sensitivity in mice
Source: Hereditas. 2025 Mar 3;162:31. doi: 10.1186/s41065-025-00377-9 (PMC11874108; doi:10.1186/s41065-025-00377-9)

***MrgF***

β-actin


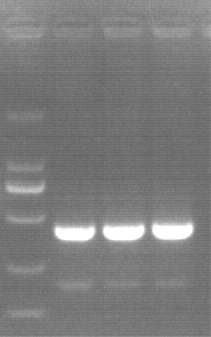


100

250

500

750

1kb

2kb


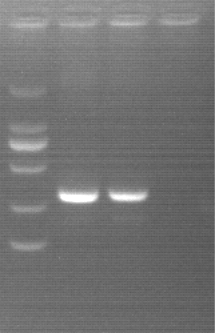


100

250

500

750

1kb

2kb


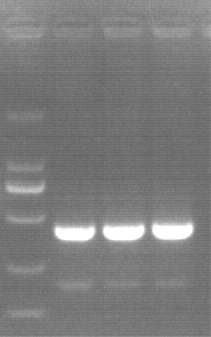

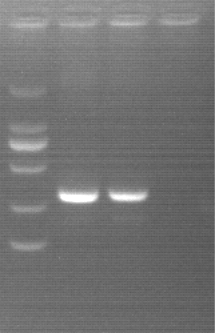


**Marker wt *MrgF+/-**MrgF-/-***

**Marker wt *MrgF+/-**MrgF-/-***

Supplement: Supplementary file 1 — Supplementary Material 1 [file 41065_2025_377_MOESM1_ESM.doc]
