# Supplementary material for: Investigating the effects of global gene knockout of MrgF on motor performance and pain sensitivity in mice
Source: Hereditas. 2025 Mar 3;162:31. doi: 10.1186/s41065-025-00377-9 (PMC11874108; doi:10.1186/s41065-025-00377-9)
Supplement: Supplementary file 2 — Supplementary Material 2 [file 41065_2025_377_MOESM2_ESM.doc]

**­­Supplemental figures and figure legends**

**Figure S1.**

**Supplementary Figure 1 (Figure S1)**

**Mouse body weight evaluation.**

Body weights of littermate syngeneic mice of the same sex were monitored and recorded weekly from weeks 4 to 10.

**Figure S2.**


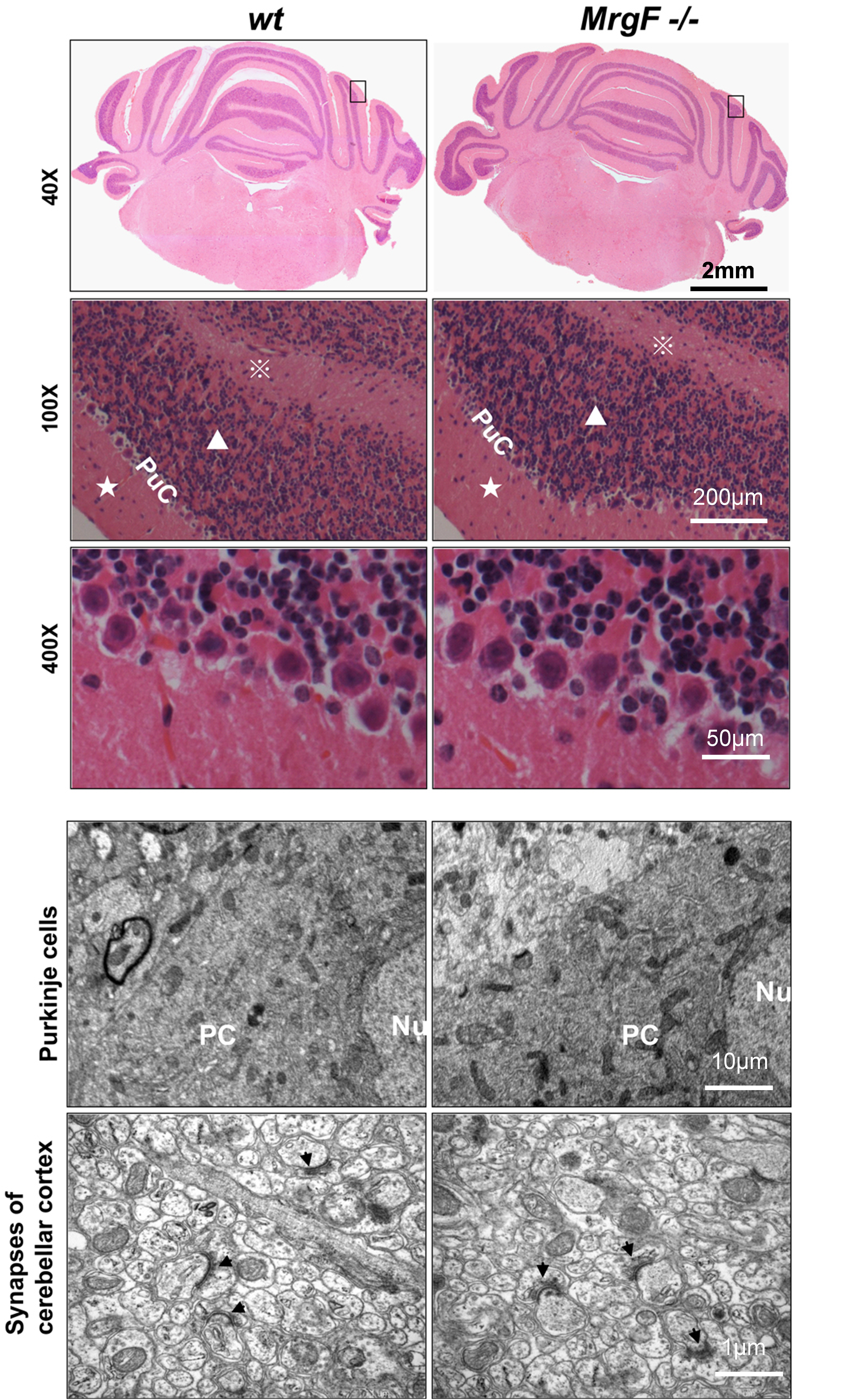


**Supplementary Figure 2 (Figure S2)**

**No significant difference in morphology and structure in the cerebellum of *MrgF* knockout mice.**

1. Histological analysis of the cerebellum in wt and *MrgF^-/-^* mice was conducted using H&E staining. Rows 2 and 3 show enlargements of the black boxes in the top row. The white star marks the molecular layer, the white triangle marks the granular layer, and the Purkinje cell layer (PuC) lies between them. The ※ indicates the cerebellar medulla. Original magnifications: ×40 (top row), ×100 (second row), ×400 (third row).
2. TEM was used to analyze the cerebellum of wt and *MrgF^-/-^* mice. The top rows show ultrastructure of Purkinje cells (PC) and nuclei (Nu), while the bottom row highlights synapse ultrastructure, with arrows indicating synapse locations. Magnifications are ×2900 (top) and ×29,500 (bottom).
